# Association of KLK4 rs2235091 polymorphism with susceptibility to dental caries: a systematic review and meta-analysis

**DOI:** 10.3389/fped.2023.1236000

**Published:** 2023-08-30

**Authors:** Youqin Li, Lei Zhang, Wen Cen, Yongping Yuan

**Affiliations:** Department of Stomatology, Ningbo College of Health Sciences, Ningbo, ZJ, China

**Keywords:** KLK4, polymorphism, meta-analysis, dental caries, caries susceptibility

## Abstract

**Objective:**

To investigate the association between Kallikrein-related peptidase-4 (KLK4) rs2235091 polymorphism and susceptibility to dental caries (DC) by a method of systematic review and meta-analysis.

**Methods:**

Four English databases were searched for studies on the correlation between KLK4 rs2235091 polymorphism and susceptibility to DC from inception to April 1, 2023. Data analysis was processed by Stata 15.0 software.

**Results:**

Four articles were eligible, including 848 individuals with caries and 463 controls. The results of pooled analysis showed no significant differences in the five gene models (G vs. A: odds ratio (OR) = 1.14, 95% CI: 0.73–1.79, *P* = 0.567; GG + GA vs. AA: OR = 1.01, 95% CI: 0.77–1.32, *P* = 0.489; GG vs. GA + AA: OR = 0.84, 95% CI: 0.57–1.23, *P* = 0.368; GA vs. AA: OR = 1.06, 95% CI: 0.80–1.41, *P* = 0.681; GG vs. AA: OR = 1.15, 95% CI: 0.57–2.31, *P* = 0.690). However, subgroup analysis indicated a statistically significant difference in the dominant (GG + GA vs. AA: OR = 1.74, 95% CI: 1.02–2.96, *P* = 0.042) gene model in primary dentition, but no significance in allelic, recessive, homozygous and heterozygous models. Besides, in permanent dentition, no significant differences were found among the five genetic models (all *P* > 0.05).

**Conclusion:**

KLK4 rs2235091 polymorphism may be associated with susceptibility to DC of pediatric primary dentition, but not with the risk of caries of permanent dentition. Genotype GG + GA may increase susceptibility to DC of pediatric primary dentition. However, considering the limited records enrolled in this review, more trials with larger sample sizes and more rigorous designs are needed to verify the conclusions of this meta-analysis in the future.

**Systematic Review Registration:**

https://inplasy.com/, identifier INPLASY202380014.

## 1. Introduction

Dental caries (DC) is a plaque-mediated disease in which chronic progressive destruction of dental hard tissue is triggered by various factors, mainly bacteria (1). Early childhood caries is defined as the presence of one or more decayed, missing, or filled teeth in children under 71 months of age (2). DC has been listed by the World Health Organization (WHO) as one of the three major human-focused diseases (3). Deciduous DC is the most common oral disease in children clinically and a serious social public health problem in developing countries (4, 5). Its prevalence in children in some developed countries is at a low level but is relatively high and dramatically on the rise in underdeveloped countries (6).

Key factors inducing the occurrence of DC include bacteria, food, time, and some necessary conditions provided by the host (6). Genetic polymorphism in the host is also a vital one affecting the development of this disease (7, 8). The human tissue kallikrein-related peptidase (KLK) gene family encodes a group of serine proteases, and 15 members have been found and named KLK1-KLK15 according to the order they are arranged on the chromosome (9). Kallikrein-related peptidase-4 (KLK4) has been largely studied in the field of stomatology and is thought to be associated with the growth and mineralization of enamel (10, 11). KLK4 is a necessary protein to clear enamel proteins and complete crystal maturation during enamel maturation, and its function is mainly played in the maturation stage of enamel development (12).

There are several existing publications reporting the relationship of susceptibility to DC with KLK4 rs2235091 gene polymorphism (13–16), but the conclusions are still controversial. Zaorska et al. (15) showed that allele G of KLK4 rs2235091 could increase the risk of DC in primary dentition. However, Gachova et al. (16) reported that the allele G at KLK4 rs2235091 locus was not associated with the DC risk in primary dentition compared with the A allele. Therefore, in this study, we used a method of systematic review and meta-analysis to explore this association and provide an evidence-based medical basis for the etiological exploration of DC.

## 2. Methods

This systematic review and meta-analysis was performed according to the guidelines of Preferred Reporting Items for Systematic Reviews and Meta-Analyses (PRISMA) (17). We have registered this trial in INPLASY (Registration number: INPLASY202380014) (https://inplasy.com/).

### 2.1. Search strategy

The PubMed, Web of Science, Cochrane Library, and Embase databases were searched for relevant English literature published from inception to April 1, 2023. Literature on the association of susceptibility to DC with KLK4 rs2235091 polymorphism was collected, and reference lists of the retrieved literature were further searched to expand our search results. The main search strategy was as follows: (“KLK4” OR “kallikrein-related peptidase-4”) AND (“dental caries” OR “caries” OR “decay”) AND (“Single nucleotide polymorphism” OR “polymorphism” OR “variant”). The search language was limited to English. In addition, we traced the relevant records through reviews and searched them manually to improve the search. The included databases were searched independently by two researchers and finally cross-checked. In this process, if there were disputes, they were resolved through discussion.

### 2.2. Inclusion and exclusion criteria

#### 2.2.1. Inclusion criteria

(1) Study design: Cross-sectional, case-control, or cohort studies on KLK4 rs2235091 polymorphism associated with deciduous tooth caries or permanent dental caries; (2) Populations: Individuals diagnosed with DC or those without DC, general good health, without age restrictions; (3) Comparison: Frequency of individuals with DC in the population without KLK4 rs2235091 polymorphism; (4) Exposure: Frequency of individuals with DC in the population with KLK4 rs2235091 polymorphism; (5) Outcomes: The rate of allele or genotype frequency according to caries incidence. The caries phenotype involved incipient or white spot lesions, and cavitated lesions.

#### 2.2.2. Exclusion criteria included

(1) Letters, conference abstracts, animal or cell experiments; (2) Articles without access to obtain full text or original data; (3) Inadequate data provided to extract the data of each genotype and calculate the pooled odds ratio (OR) of each genetic model, or literature directly not reporting the corresponding OR value in the genetic model.

### 2.3. Data extraction

Two investigators independently performed data extraction and cross-checked them. Then they extracted relevant data from the included articles: first author, publication time, region of subjects, study design, sample size, ages of DC group, diagnostic criteria for DC, genotyping methods, dentition type, and number of genotypes in DC and caries free, or OR value in the genetic model at KLK4 rs2235091 locus.

### 2.4. Literature quality evaluation

The methodological qualities of the cohort and case-control studies were assessed using the NOS score (18). The overall scores were 9. Scores of 7–9, 4–6, and <4 indicated high, moderate, and low methodological quality, respectively.

The cross-sectional study was assessed by the evaluation criteria recommended by the Agency for Healthcare Research and Quality (AHRQ) (19). The AHRQ criteria contained 11 items in total, and each item had 3 options; “Yes” counted 1 score, while “No” or “Unclear” counted 0 scores. According to the total scores, the papers were rated as low-quality research (0–3 scores), medium-quality research (4–7 scores), or high-quality research (8–11 scores) (20).

### 2.5. Statistical analysis

Stata 15.0 statistical software was applied for this meta-analysis. ORs and 95% confidence intervals (CIs) served as effect sizes. We calculated the Hardy-Weinberg Equilibrium in the controls. Heterogeneity across the included studies was assessed using the *χ*^2^ test and *I*^2^ statistics. *P* > 0.05 and *I*^2^ < 50% pointed to no significant heterogeneity, and a fixed-effects model (FEM) was utilized; otherwise, a random-effects model (REM) was employed. Subgroup analysis was performed according to whether the caries were primary or permanent dentition. We adopted Egger's test to inspect publication bias and sensitivity analyses to verify the stability of the results obtained.

## 3. Results

### 3.1. Literature screening results and general characteristics

**Figure 1:**
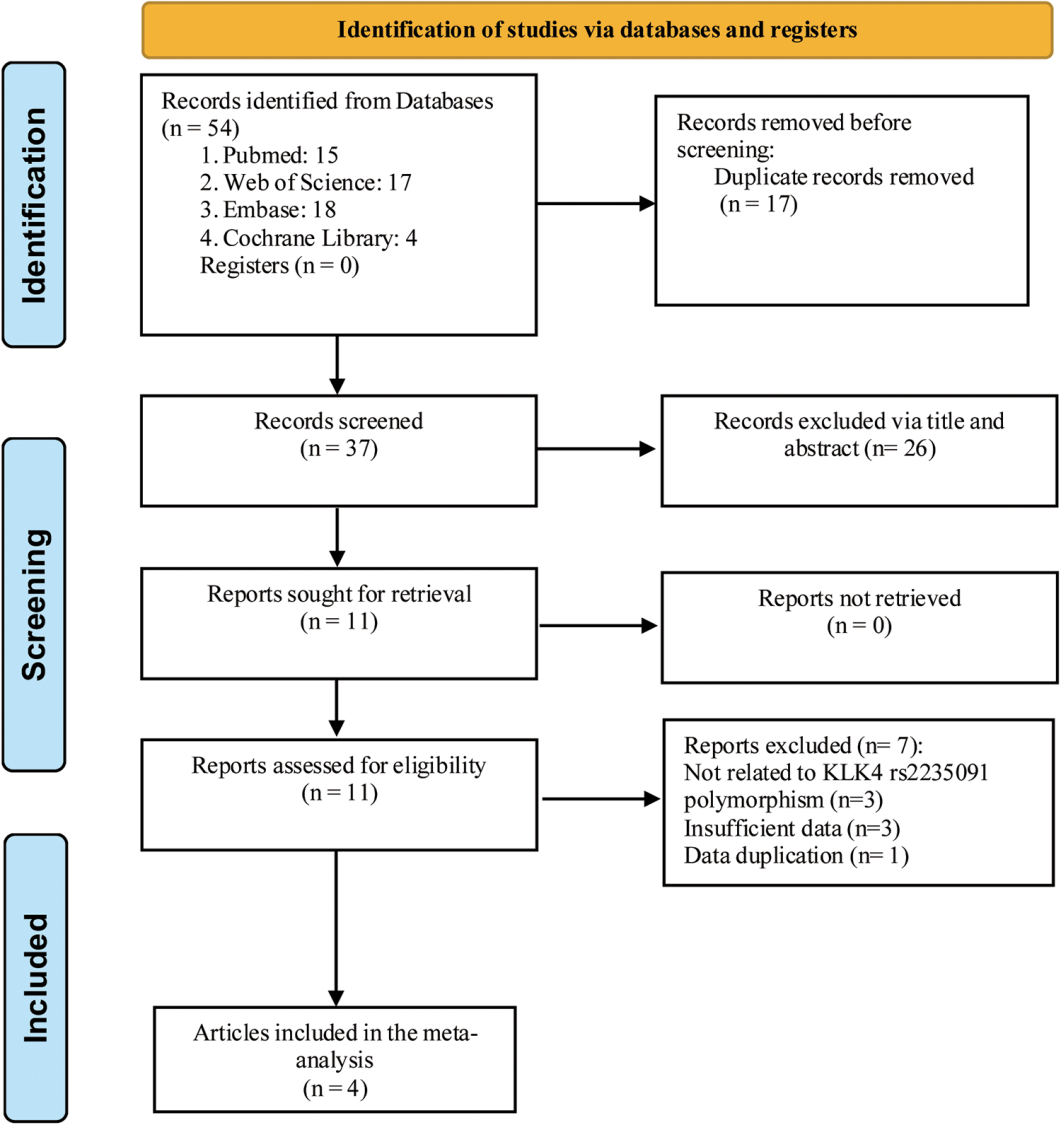
Flow chart of literature retrieval.

Four articles were entered into our meta-analysis (13–16) (Figure 1), involving 848 individuals with caries and 463 controls. Among the four articles included, one article contained two studies (16). Besides, one article did not report the number per genotype in the cases and control group, but reported the corresponding OR values in both homozygous (GG vs. AA: OR = 1.58, 95% CI: 0.38–6.55) and heterozygous (GA vs. AA: OR = 1.58, 95% CI: 0.38–6.55) models (13). The basic characteristics of the included records are illustrated in Table 1. The NOS scores were all above 6, and the AHRQ scores were all more than 7, indicating the high quality of the included records.

**Table 1:** Basic characteristics of studies included in this meta-analysis.

Study	Year	Country	Study design	Detection method	Dentition type	Number	DMFT (DC/Con)	Ages of DC group	Caries experience			Caries free			HWE	NOS/AHRQ
						(DC/Con)			GG	GA	AA	GG	GA	AA		
Abbasoğlu (13)	2015	Turkey	CSS	PCR	Primary	136/123	≥1/0	2–5(years)	NR	NR	NR	NR	NR	NR	N/A	8
Cavallari (14)	2017	Brazil	CSS	PCR	Permament	97/99	≥1/0	12–34(years)	7	56	34	8	52	39	0.101	9
Zaorska (15)	2021	Poland	CCS	PCR	Primary	48/47	≥1/0	20–40(month)	10	17	21	4	11	32	0.060	7
Gachova (16)	2022	Czech Republic	CCS	RFLP-PCR	Primary	105/45	≥10/0	2–6(years)	21	43	41	9	16	20	0.102	8
					Permament	462/149	≥1/0	13–15(years)	49	198	215	25	67	57	0.485	

Con, control; N/A, not applicable; PCR, polymerase chain reaction; RFLP, restriction fragment length polymorphism; DMFT, decayed, missing, and filled teeth; NOS, Newcastle-Ottawa scale; NR: not reported; HWE, Hardy-Weinberg equilibrium; DC, dental caries; CSS, cross-sectional study; CCS, case–control study; AHRQ, Agency for Healthcare Research and Quality.

### 3.2. Relationship between KLK4 rs2235091 polymorphism and susceptibility to DC

**Figure 2:**
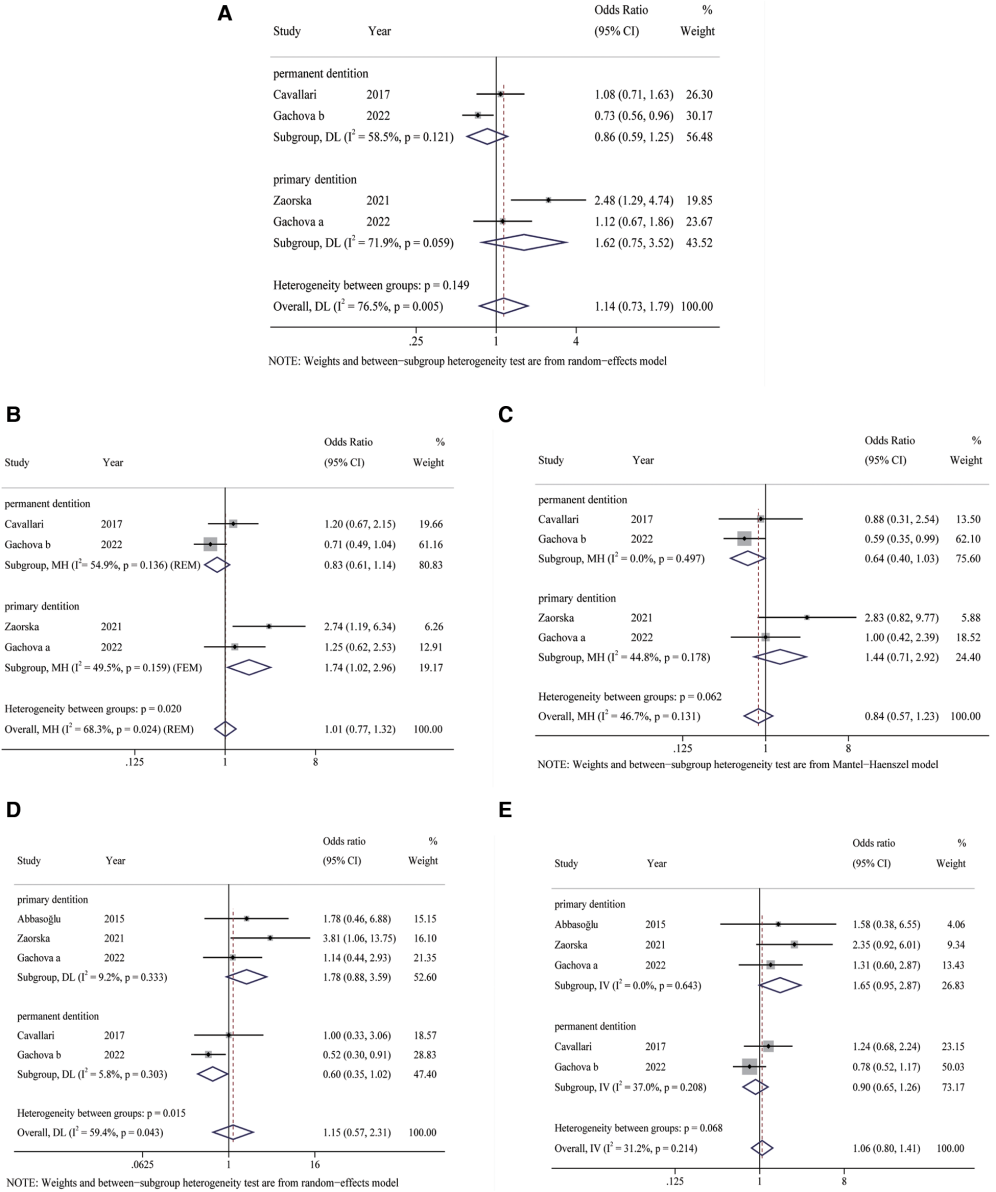
Forest plots of KLK4 rs2235091 polymorphism with risk of dental caries. (**A**) Allele model; (**B**): dominant model; (**C**): recessive model; (**D**): homozygous model; (**E**): heterozygous model. FEM, fixed-effects model; REM, random-effects model.

No significant heterogeneity was observed in the heterozygous (*I*^2^ = 31.2%, *P* = 0.214) and recessive (*I*^2^ = 46.7%, *P* = 0.131) genetic models, so the FEM was selected for statistical analysis. The REM was applied for analysis of allelic (*I*^2^ = 76.5%, *P* = 0.005), dominant (*I*^2^ = 68.3%, *P* = 0.024), and homozygous (*I*^2^ = 59.4%, *P* = 0.043) gene models because of significant heterogeneity (Figures 2A–E).

The results of the meta-analysis (Figure 2A–E) showed no significant differences in the five genetic models (G vs. A: OR = 1.14, 95% CI: 0.73–1.79, *P* = 0.567; GG + GA vs. AA: OR = 1.01, 95% CI: 0.77–1.32, *P *= 0.489; GG vs. GA + AA: OR = 0.84, 95% CI: 0.57–1.23, *P *= 0.368; GA vs. AA: OR = 1.06, 95% CI: 0.80–1.41, *P* = 0.681; GG vs. AA: OR = 1.15, 95% CI: 0.57–2.31, *P *= 0.690).

Results of subgroup analysis displayed statistically significant differences in the dominant (GG + GA vs. AA: OR = 1.74, 95% CI: 1.02–2.96, *P* = 0.042) gene models in caries of primary dentition (Figure 2B). Nevertheless, no statistically significant differences were found in the allelic (G vs. A: OR = 1.62, 95% CI: 0.75–3.52, *P* = 0.222), recessive (GG vs. GA + AA: OR = 1.44, 95% CI: 0.71–2.92, *P *= 0.312), homozygous (GG vs. AA: OR = 1.78, 95% CI: 0.88–3.59, *P *= 0.108) and heterozygous (GG vs. AA: OR = 1.65, 95% CI: 0.95–2.87, *P *= 0.076) models (Figures 2A,C–E). In addition, heterogeneity was significantly reduced in dominant (I^2^ = 49.5%, *P* = 0.159), and homozygous (*I*^2^ = 5.8%, *P* = 0.303) gene models in caries of primary dentition. However, in caries of permanent dentition, no significant differences were observed in the five gene models (all *P* > 0.05) (Figures 2A–E). Therefore, KLK4 rs2235091 polymorphism is associated with susceptibility to DC of pediatric primary dentition, but not with the risk of caries of permanent dentition.

### 3.4. Sensitivity analysis

**Figure 3:**
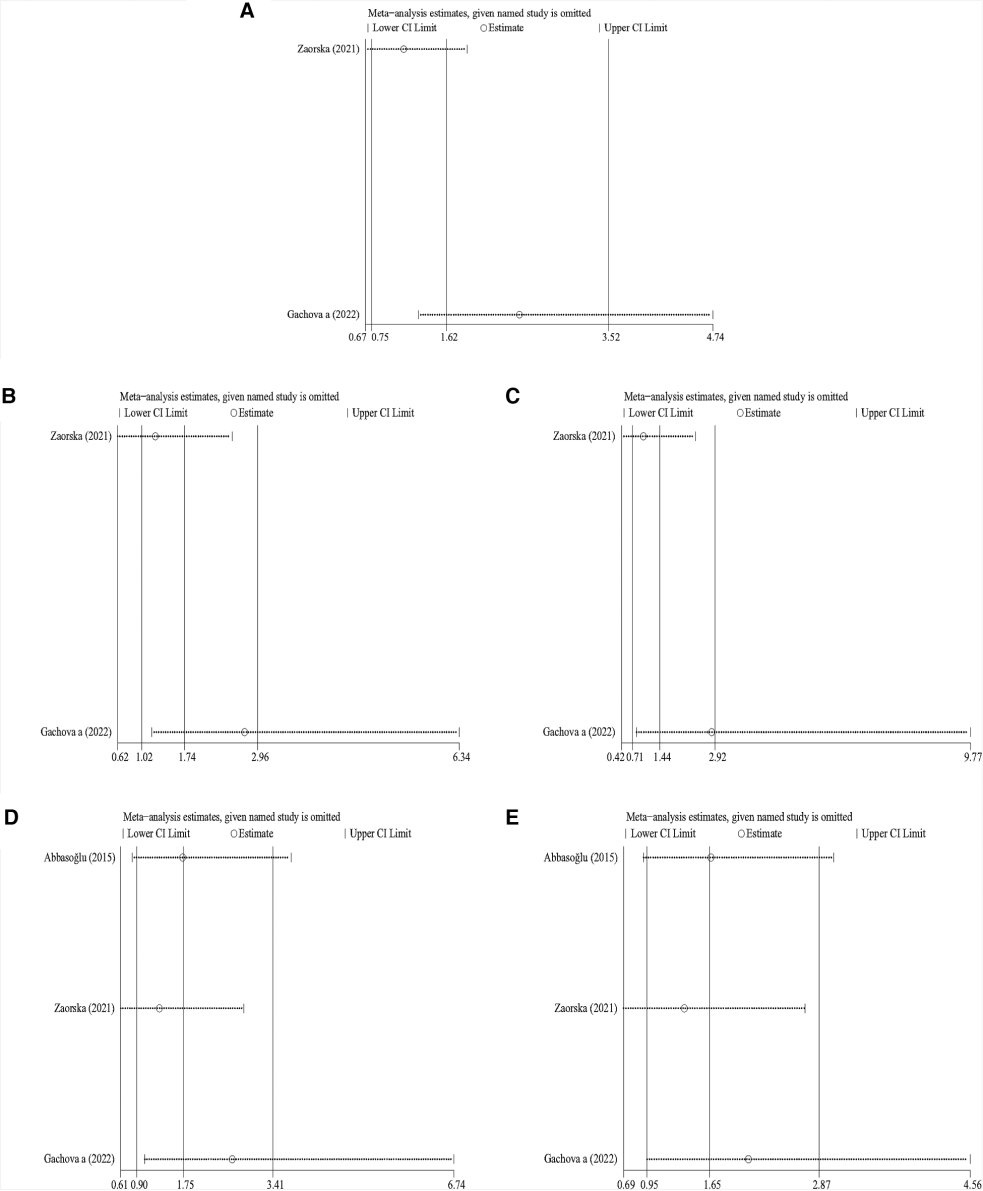
Sensitivity analysis of KLK4 rs2235091 related to caries of primary dentition. (**A**) Allele model; (**B**): dominant model; (**C**): recessive model; (**D**): homozygous model; (**E**): heterozygous model.

Sensitivity analysis was performed on the results of primary dentition. The pooled results showed that, except in the recessive and heterozygous models, differences changed significantly after the removal of one article (13), one article (15), and one article (16) in the allelic, dominant, and homozygous genetic models, respectively (Figures 3A–E). This suggested that the findings obtained in the primary dentition had instability in this meta-analysis.

### 3.5. Publication bias

The *P* values of Egger's test were 0.04, 0.106, 0.04, 0.076, and 0.690 in the allelic, recessive, dominant, heterozygous, and homozygous gene models, respectively. Therefore, a certain degree of publication bias existed in this review.

## 4. Discussion

In this study, we selected the method of systematic review and meta-analysis to inspect the association of susceptibility to DC with KLK4 rs2235091 polymorphism. In the overall meta-analysis, we did not find an association between KLK4 rs2235091 polymorphism and susceptibility to DC. It was worth noting that there were significant heterogeneities in all gene models except the recessive and heterozygote models. Thus, we performed a subgroup analysis based on whether DC was primary or permanent dentition to explore the sources of heterogeneity.

The subgroup analysis displayed that there was a significant decrease in heterogeneity under the dominant, and homozygous models in DC of primary dentition. Moreover, the results indicated an association of the KLK4 rs2235091 polymorphism with susceptibility to DC of pediatric primary dentition, but not with the risk of caries of permanent dentition. In the analysis of the primary dentition, the difference was statistically significant in the dominant gene model. In other words, compared to genotype AA at KLK4 rs2235091 locus, genotype GG + GA can increase susceptibility to DC in the primary dentition. However, we did not find statistically significant differences in allele, recessive, homozygous, and heterozygous models. Wang et al.’s study supported the protective effect of allele A at KLK4 rs2235091 locus, while allele G could increase caries risk for phenotype d2fs-smooth surface. In Wang et al.'s research (20), the mean and standard deviation of age of the subjects was 5.28 ± 0.4 years. The above finding of Wang et al. (20) is inconsistent with our conclusion that allele G was not associated with susceptibility to DC compared to allele A in primary dentition in this meta-analysis. Wang et al.'s study did not provide the frequency distribution of alleles in the control group, which restricted us from combining the results of their alleles with the allele genes in our meta-analysis. This can also be seen from the sensitivity analysis. Sensitivity analysis showed that the difference in allelic model was significant after removing one article. Therefore, more studies are needed to confirm the allelic conclusions in the future.

Due to the small number of literature included in this meta-analysis, Egger's Test was adopted to detect the possible publication bias. The results showed that although the *P* values of Egger's Test were greater than 0.05 in the recessive, homozygous, and heterozygous models, the *P* values were less than 0.05 in both the allelic and dominant gene models, suggesting that there was a certain publication bias in this meta-analysis. In the sensitivity analysis of primary dentition, except the recessive and heterozygote models, the findings among the other three gene models were not robust to some extent.

KLK4 expression is not found in ameloblasts. However, it can be detected in the glaze matrix during the secretory stage, and the content increases sharply during the transition stage and persists until the end of the maturation stage (21). According to Northern hybridization analysis, the change of KLK4 was in parallel with its mRNA content (22). Therefore, the polymorphism of KLK4 rs2235091 gene may affect the expressions of KLK4 in enamel, and thus influence the development and maturation of enamel.

Inevitably, this meta-analysis had several limitations. First, few studies were eligible, with only four articles entered into this meta-analysis. Second, the sample size was small, which might have a certain influence on the robustness of the conclusions. Third, only studies published in English were included, while possible high-quality studies published in other languages or unpublished were excluded, which might lead to a certain publication bias. Fourth, sensitivity analysis showed that the conclusions of this study are not robust. Fifth, no further analysis of gene-gene and gene-environment interactions was performed. Single polymorphism may have a minor effect, and polymorphisms across the genes may alter the susceptibility. The interactions between genes and genes, as well as between genes and environment, may jointly determine the phenotypes of DC. However, the current limited information restricts our further research. Sixth, The *P*-values of Egger's Test in the allelic and dominant gene models are less than 0.05, indicating a certain publication bias, which might have a certain impact on the robustness of the overall and subgroup analysis results. Seventh, the experimental design included in this study included case-control studies and cross-sectional investigations, which might cause heterogeneity. Nevertheless, the limited amount of literature limited our further analysis. Despite these limitations, this is the first systematic review and meta-analysis of KLK4 rs2235091 polymorphism with susceptibility to DC. Although the conclusions are not robust to some extent, they can be confirmed by further studies, which has a positive significance for exploring the etiology of DC.

In conclusion, KLK4 rs2235091 polymorphism may be associated with susceptibility to DC of pediatric primary dentition, but not with the risk of caries of permanent dentition. Genotype GG + GA may increase susceptibility to DC of pediatric primary dentition. Given that several limitations existed in this review, such as the number of included studies and small sample size, the findings of this meta-analysis need to be verified by more researches in the future.

## Data Availability

The raw data supporting the conclusions of this article will be made available by the authors, without undue reservation.

## References

[1] Kammoun R Zmantar T Labidi A Abbes I Mansour L Ghoul-Mazgar S. Dental caries and hypoplastic amelogenesis imperfecta: clinical, structural, biochemical and molecular approaches. Microb Pathog. (2019) 135:103615. 10.1016/j.micpath.2019.103615 31254603

[2] Olszowski T Adler G Janiszewska-Olszowska J Safranow K Kaczmarczyk M. MBL2, MASP2, AMELX, and ENAM gene polymorphisms and dental caries in Polish children. Oral Dis. (2012) 18(4):389–95. 10.1111/j.1601-0825.2011.01887.x 22221294

[3] Drummond BK Meldrum AM Boyd D. Influence of dental care on children’s oral health and wellbeing. Brit Dent J. (2013) 214(11):E27. 10.1038/sj.bdj.2013.533 23744240

[4] Siddiqui AA Alshammary F Mulla M Al-Zubaidi SM Afroze E Amin J Prevalence of dental caries in Pakistan: a systematic review and meta-analysis. BMC Oral Health. (2021) 21(1):450. 10.1186/s12903-021-01802-x 34530810 PMC8447584

[5] Hu J Jiang W Lin X Zhu H Zhou N Chen Y Dental caries status and caries risk factors in students ages 12–14 years in Zhejiang, China. Med Sci Monit. (2018) 24:3670–78. 10.12659/MSM.907325 29856733 PMC6007515

[6] Elamin A Garemo M Mulder A. Determinants of dental caries in children in the Middle East and North Africa region: a systematic review based on literature published from 2000 to 2019. BMC Oral Health. (2021) 21(1):237. 10.1186/s12903-021-01482-7 33947387 PMC8097819

[7] Wu L Li Z Zhou J Ma B Yu F Zheng X An association analysis for genetic factors for dental caries susceptibility in a cohort of Chinese children. Oral Dis. (2022) 28(2):480–94. 10.1111/odi.13758 33345418

[8] Chisini LA Cademartori MG Conde MCM Tovo-Rodrigues L Correa MB. Genes in the pathway of tooth mineral tissues and dental caries risk: a systematic review and meta-analysis. Clin Oral Invest. (2020) 24(11):3723–38. 10.1007/s00784-019-03146-x 32945961

[9] Dorn J Beaufort N Schmitt M Diamandis EP Goettig P Magdolen V. Function and clinical relevance of kallikrein-related peptidases and other serine proteases in gynecological cancers. Crit Rev Clin Lab Sci. (2014) 51(2):63–84. 10.3109/10408363.2013.865701 24490956

[10] Núñez SM Chun YP Ganss B Hu Y Richardson AS Schmitz JE Maturation stage enamel malformations in amtn and Klk4 null mice. Matrix Biol. (2016):219–33. 10.1016/j.matbio.2015.11.007 26620968 PMC4875837

[11] Hu Y Smith CE Richardson AS Bartlett JD Hu JC Simmer JP. MMP20, KLK4, and MMP20/KLK4 double null mice define roles for matrix proteases during dental enamel formation. Mol Genet Genom Med. (2016) 4(2):178–96. 10.1002/mgg3.194 PMC4799876 27066511

[12] Simmer JP Hu Y Lertlam R Yamakoshi Y Hu CC. Hypomaturation enamel defects in Klk4 knockout/LacZ knockin mice. J Biol Chem. (2009) 284(28):19110. 10.1074/jbc.M109.013623 19578120 PMC2707199

[13] Abbasoğlu Z Tanboğa Ï Küchler EC Deeley K Weber M Kaspar C Early childhood caries is associated with genetic variants in enamel formation and immune response genes. Caries Res. (2015) 49(1):70–7. 10.1159/000362825 25531160 PMC4376372

[14] Cavallari T Tetu Moyses S Moyses SJ Iani Werneck R. KLK4 Gene and dental decay: replication in a south Brazilian population. Caries Res. (2017) 51(3):240–43. 10.1159/000464450 28445870

[15] Zaorska K Szczapa T Borysewicz-Lewicka M Nowicki M Gerreth K. Prediction of early childhood caries based on single nucleotide polymorphisms using neural networks. Genes (Basel). (2021) 12(4):462. 10.3390/genes12040462 33805090 PMC8064067

[16] Gachova D Lipovy B Deissova T Izakovicova Holla L Danek Z Borilova Linhartova P. Polymorphisms in genes expressed during amelogenesis and their association with dental caries: a case-control study. Clin Oral Invest. (2022). 10.1007/s00784-022-04794-2 PMC10102052 36422720

[17] Moher D Liberati A Tetzlaff J Altman DG. Preferred reporting items for systematic reviews and meta-analyses: the PRISMA statement. Int J Surg. (2010) 8(5):336–41. 10.1016/j.ijsu.2010.02.007 20171303

[18] Stang A. Critical evaluation of the Newcastle-Ottawa scale for the assessment of the quality of nonrandomized studies in meta-analyses. Eur J Epidemiol. (2010) 25(9):603–05. 10.1007/s10654-010-9491-z 20652370

[19] Rostom A Dubé C Cranney A Saloojee N Sy R Garritty C Celiac disease. Rockville, MD: Agency for Healthcare Research and Quality (US) (2004). (Evidence Reports/Technology Assessments, No. 104.) Appendix D. Quality Assessment Forms. Available at: https://www.ncbi.nlm.nih.gov/books/NBK35156/

[20] Feng X Tang Q Cheng C Xu S. Low serum lipid levels, use of statin and cerebral microbleeds: a systematic review and meta-analysis. J Clin Neurosci. (2021) 94:216–25. 10.1016/j.jocn.2021.10.032 34863441

[21] Wang X Willing MC Marazita ML Wendell S Warren JJ Broffitt B Genetic and environmental factors associated with dental caries in children: the Iowa fluoride study. Caries Res. (2012) 46(3):177–84. 10.1159/000337282 22508493 PMC3580152

[22] Hu CC Zhang C Sun X Yang Y Cao X Ryu O Characterization of the mouse and human PRSS17 genes, their relationship to other serine proteases, and the expression of PRSS17 in developing mouse incisors. Gene. (2000) 251(1):8. 10.1016/s0378-1119(00)00203-1 10863090

[23] Scully JL Bartlett JD Chaparian MG Fukae M Uchida T Xue J Enamel matrix serine proteinase 1: stage-specific expression and molecular modeling. Connect Tissue Res. (1998) 39:111–22. 10.3109/03008209809023917 11062993

